# Adaptive Palliative Stereotactic Body Radiotherapy for a Large Cervical Lymph Node Metastasis in a Near-Centenarian Patient: A Case Report

**DOI:** 10.7759/cureus.113392

**Published:** 2026-07-26

**Authors:** Shinichiro Mizumatsu, Shohei Nambu, Fumiya Baba, Morikazu Hasegawa, Tsuyoshi Satoh

**Affiliations:** 1 Department of Radiology, Narita Memorial Hospital, Toyohashi, JPN; 2 Home Care, Alpaca Family Clinic, Toyohashi, JPN; 3 Department of Palliative Care, Narita Memorial Hospital, Toyohashi, JPN

**Keywords:** adaptive radiotherapy (art), cone-beam computed tomography (cbct), home-care physician, image-guided radiotherapy (igrt), interdisciplinary collaboration, near-centenarian, pain relief, palliative radiotherapy, sbrt (stereotactic body radiotherapy), very-elderly

## Abstract

Palliative radiotherapy for very-elderly patients requires rapid symptom relief, short treatment times, fewer hospital visits, and minimal side effects. We report a 99-year-old woman referred to our department by her home-care physician due to severe pain from a rapidly growing, large cervical lymph node metastasis. The initial plan was stereotactic body radiotherapy (SBRT) with a prescribed dose of 24 Gy in three fractions over seven days. Her pain decreased rapidly after the first fraction. Before the third fraction, pre-treatment cone-beam computed tomography for image-guided radiotherapy showed significant tumor shrinkage, with complete resolution of local pain. To minimize the risk of toxicities, we made an adaptive decision to cancel the third fraction. Ultimately, the treatment was completed with a prescribed dose of 16 Gy in two fractions over four days, without any adverse events. The patient passed away peacefully due to old age 56 days after initiating SBRT, maintaining complete pain control until death. Palliative SBRT incorporating adaptive radiotherapy based on intra-treatment imaging and clinical presentation is an effective and safe option for extremely elderly patients. It is important to raise awareness of this low-risk, high-precision treatment in community and palliative care settings.

## Introduction

In recent years, although the overall prognosis for cancer patients has improved, elderly individuals are often excluded from clinical trials. Consequently, establishing evidence-based treatment strategies for this population remains challenging [1]. Palliative radiotherapy (PR) for elderly patients requires minimal invasiveness, rapid symptom relief, short overall treatment times, brief single-fraction irradiation times, and a low incidence of adverse events [2-4]. In contrast, long-term local control, the prevention of new lesions, and late adverse events are often secondary concerns in palliative settings of "Best Supportive Care", where maintaining immediate quality of life (QOL) is paramount [5]. Stereotactic body radiotherapy (SBRT) meets these requirements by delivering high radiation doses precisely from multiple directions [6]. This approach maximizes therapeutic efficacy while minimizing exposure to surrounding normal tissues, thereby reducing acute toxicity. Furthermore, while conventional radiotherapy (CRT) typically spans several weeks, SBRT can be completed within a few days [2,6]. Because it is significantly less invasive than surgery, SBRT represents a highly viable and effective option for palliative care in elderly patients [2-4,6]. Recent technological innovations have further enhanced the utility of SBRT. Flattening filter-free (FFF) beams and volumetric modulated arc therapy (VMAT) have substantially reduced the irradiation time per fraction [7]. For precise localization, thermoplastic immobilization devices, body immobilization systems, and image-guided radiotherapy (IGRT) using cone-beam computed tomography (CBCT) allow high-precision targeting in the neck and trunk [8]. More recently, adaptive radiotherapy (ART) has gained considerable attention. ART dynamically optimizes the treatment plan or delivery schedule in response to morphological or biological changes in tumors or organs during the treatment course [9]. In this case report, we describe a 99-year-old patient with cervical lymph node metastasis who underwent palliative SBRT utilizing ART, which successfully achieved favorable symptom relief without significant toxicities. In this case, the ART approach involved omitting a scheduled fraction rather than modifying the treatment plan, as intra-treatment imaging and rapid symptom resolution confirmed that the palliative intent had been achieved.

## Case presentation

**Figure 1:**
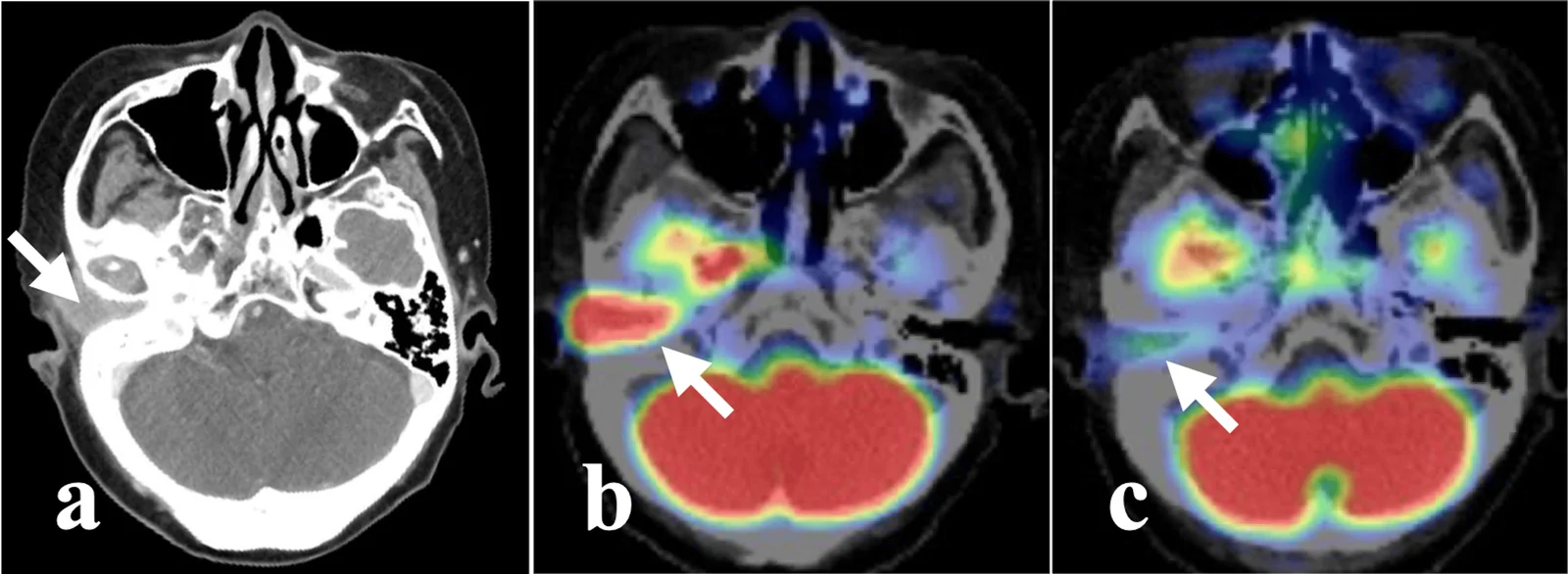
CT images of the patient showing right external auditory canal cancer (a, b) Pre-treatment images. (a) Contrast-enhanced axial CT showing an enhancing right external auditory canal lesion (white arrow); (b) Axial FDG-PET/CT demonstrating intense FDG uptake in the right external auditory canal lesion (white arrow); (c) Axial FDG-PET/CT obtained six months after CRT, showing complete regression of the right external auditory canal lesion and resolution of the abnormal FDG uptake (white arrow). CT; computed tomography, CRT; conventional radiotherapy, FDG; 2-deoxy-2-[¹⁸F]fluoro-D-glucose, PET; positron emission tomography.

The patient was a 99-year-old woman. At age 98, she developed right external auditory canal cancer (Figures 1a, 1b).

Biopsy and pathological examination revealed invasive proliferation of poorly differentiated cancer cells. On immunohistochemistry examination, the tumor cells were found positive for cytokeratin (+/-), CK7 (+), c-kit (CD117) (+), and p53 (+); negative for CK20 (-), CK5/6 (-), p63 (-), vimentin (-), and chromogranin A (-); and partially positive for synaptophysin (+/-) and insulinoma-associated protein 1 (+/-). The Ki-67 labeling index was high. Although a definitive histological diagnosis was challenging, poorly differentiated ceruminous gland carcinoma and sebaceous gland carcinoma were the primary differential diagnoses. She underwent CRT (66 Gy in 33 fractions) targeting the primary lesion, which led to complete tumor regression (Figure 1c). Ten months after this primary treatment, swelling appeared in the left neck. Lymph node biopsy revealed numerous clusters of atypical epithelial cells with enlarged nuclei and increased chromatin, against a background of lymphocytes. The clusters exhibited irregular overlapping. It was diagnosed as metastatic carcinoma. Given her advanced age, a best supportive care approach was initially adopted, and home-based medical care was initiated. However, as the tumor enlarged, local pain, erythema, and swelling progressively worsened. Consequently, 13 months after the completion of CRT, she was referred to our department by her home-care physician for palliative symptom relief.

**Figure 2:**
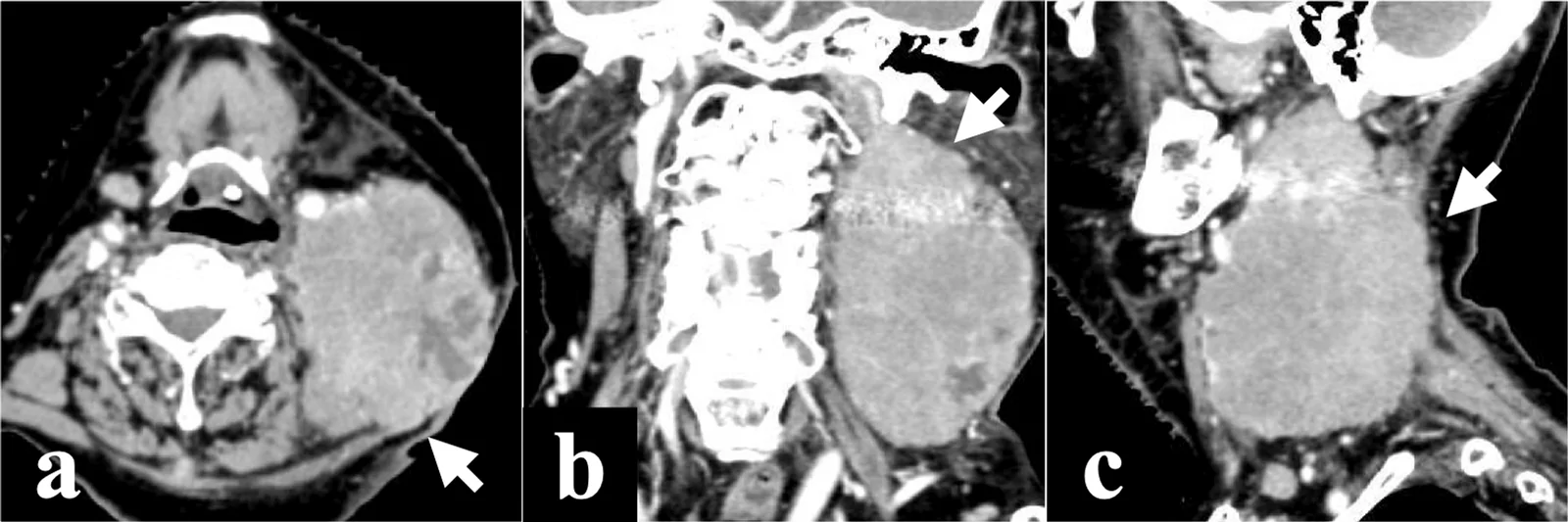
Contrast-enhanced CT images of the patient's neck Contrast-enhanced CT demonstrating the left cervical lymph node metastasis (measuring 10 × 8 cm) before SBRT (white arrow). (a) Axial, (b) coronal, and (c) sagittal views. CT; computed tomography, SBRT; stereotactic body radiotherapy.

At her initial presentation, the patient had hearing loss but lived alone and was fully independent in her activities of daily living. Her Eastern Cooperative Oncology Group performance status was 2, and her Karnofsky Performance Status was 70%. She had a strong family support system, with relatives residing nearby. To manage her symptoms, her home-care physician had prescribed opioids and betamethasone. Physical examination revealed a large, painful mass in the left neck, without skin ulceration or active bleeding. Contrast-enhanced CT demonstrated a large metastatic tumor in the left neck (Figure 2). 

**Figure 3:**
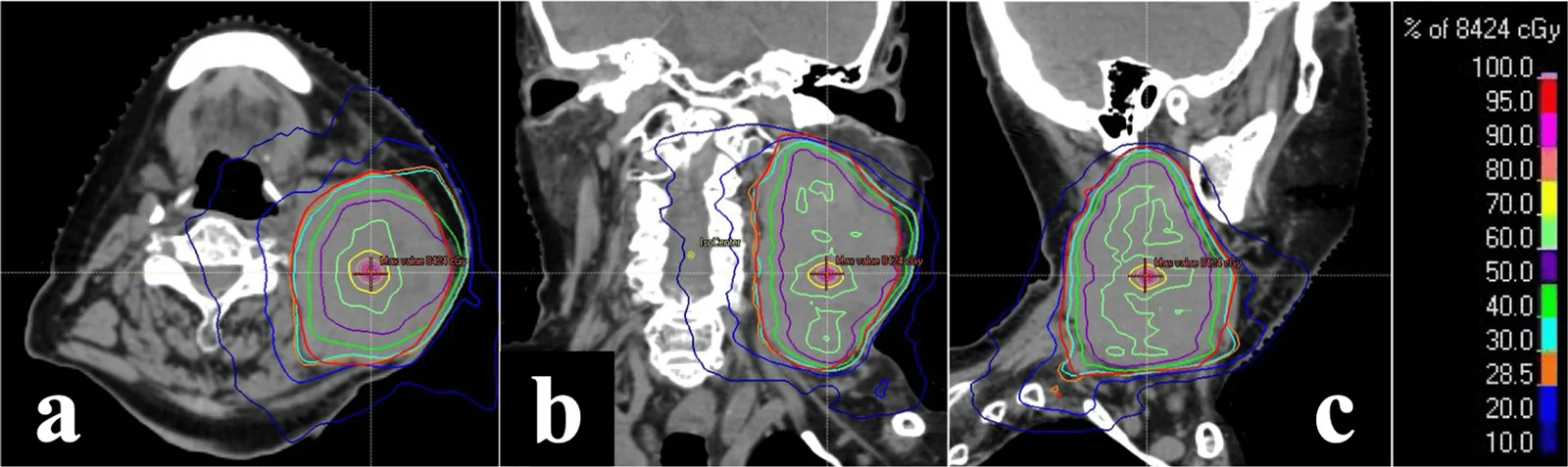
Initially planned dose distribution of SBRT Isodose lines are displayed relative to the maximum dose (100% = 84.24 Gy) on non-contrast CT. (a) Axial, (b) coronal, and (c) sagittal views. Irradiation time is 304 seconds per fraction via three arcs at O-ring angles of 0°, 20°, and 340° using the OXRAY® system (Hitachi High-Tech Corporation, Tokyo, Japan). SBRT; stereotactic body radiotherapy, CT; computed tomography.

On the same day, an immobilization device was molded, and a planning CT scan was acquired. The SBRT plan was designed with a prescription dose (the dose covering 95% of the target volume; D95) of 24 Gy in three fractions over seven days, with a maximum dose of 84.24 Gy (Figure 3).

**Figure 4:**
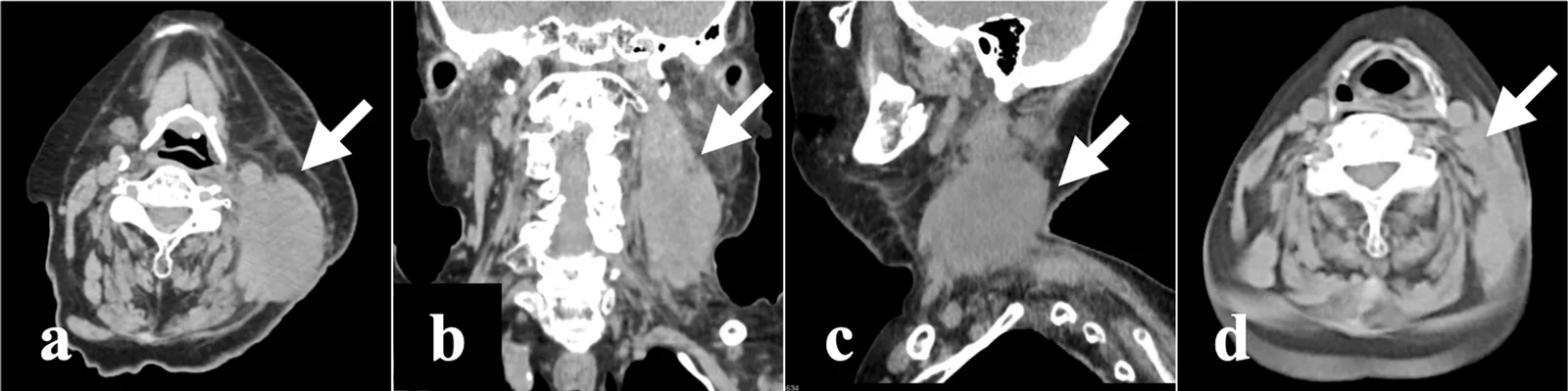
Non-contrast CT images of the patient's neck The images are showing the left cervical lymph node metastasis following SBRT: (a–c) non-contrast CT obtained on day 7 (the scheduled third fraction), showing initial tumor regression (white arrow). (a) Axial, (b) coronal, and (c) sagittal views. (d) Axial non-contrast CT obtained 37 days after SBRT, showing further tumor regression (white arrow). CT; computed tomography, SBRT; stereotactic body radiotherapy.

The cumulative dose from the initial CRT could not be calculated because it was delivered at a different facility. Although a metastasis to the left supraclavicular lymph node was also detected, it was excluded from the target volume because it was asymptomatic. SBRT was initiated two days after the initial consultation using an O-ring linear accelerator, OXRAY® (Hitachi High-Tech Corporation, Tokyo, Japan). Irradiation was delivered via VMAT using 6MV-FFF beams. The O-ring gantry angles were set at 0°, 20°, and 340°, and three arcs were used for delivery. The total irradiation time was 304 seconds per fraction. Her local pain rapidly subsided after the first fraction, allowing her to sleep comfortably. Pre-treatment CBCT for IGRT on day 4 (the second fraction) demonstrated tumor shrinkage. Pre-treatment CBCT for IGRT on day 7 (the scheduled third fraction) revealed an even more pronounced reduction in tumor volume (Figures 4a-4c), at which point her local symptoms had completely resolved. 

Considering that the primary objective of palliation had been fully achieved and the tumor exhibited marked radiosensitivity, the third fraction was canceled, and the treatment was terminated. Ultimately, the delivered dose after omitting the third fraction was a total prescribed dose (D95) of 16 Gy and a maximum dose of 56.2 Gy, delivered in two fractions over four days. The maximum doses received in the two sessions were 10.08 Gy to the brainstem, 17.11 Gy to the esophagus, pharynx, and larynx, 19.38 Gy to the left parotid gland, and 23.55 Gy to the skin surface.

**Figure 5:**
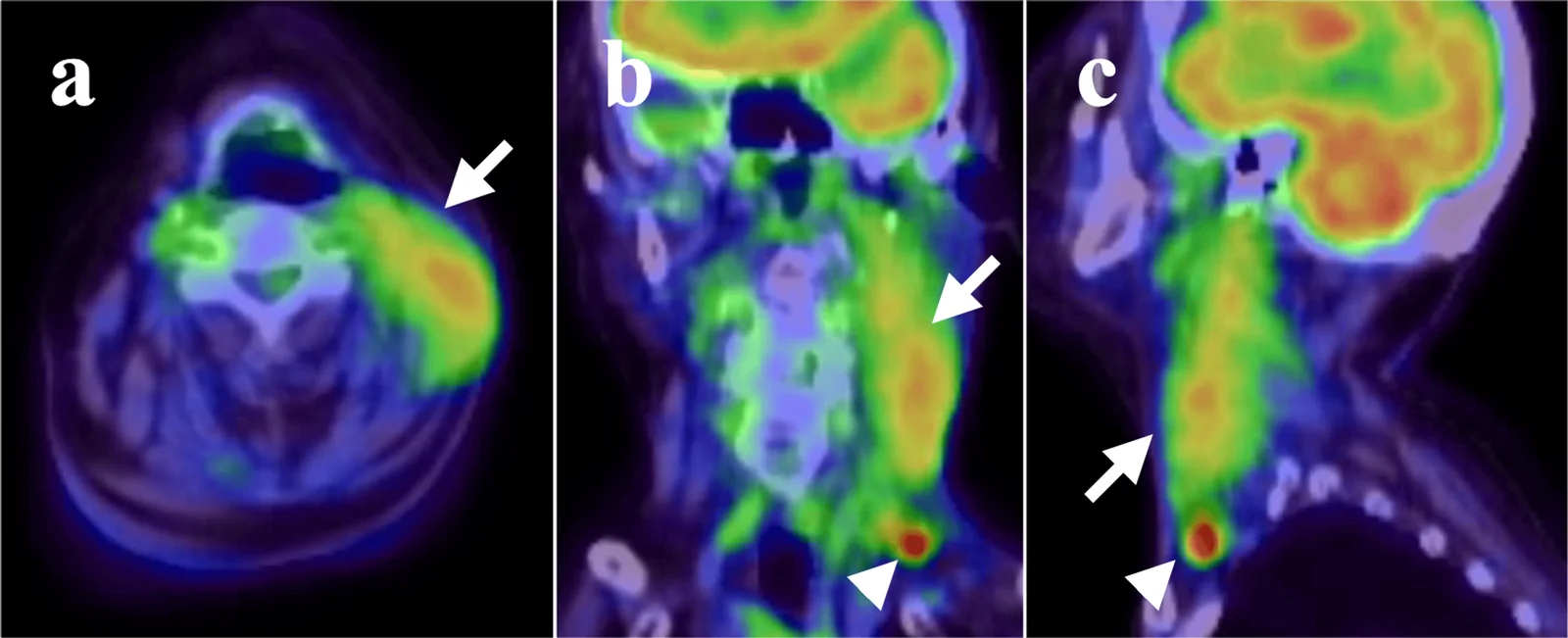
FDG-PET/CT images of the patient's neck obtained nine days after SBRT (a–c) The left cervical lymph node metastasis showing moderate FDG uptake (white arrow). (a) Axial, (b) coronal, and (c) sagittal views. (b, c) An unirradiated left supraclavicular lymph node metastasis showing intense FDG uptake (white arrowhead). FDG; 2-deoxy-2-[¹⁸F]fluoro-D-glucose, PET; positron emission tomography, CT; computed tomography, SBRT; stereotactic body radiotherapy.

Direct contralateral lymph node metastasis from head and neck cancer is extremely rare. Given this atypical clinical course and the marked radiosensitivity, we considered alternative diagnoses, such as a hematological malignancy. Therefore, 2-deoxy-2-[¹⁸F]fluoro-D-glucose (FDG) positron emission tomography/CT was performed nine days after the final SBRT fraction. However, no significant FDG uptake was detected outside the left neck and supraclavicular lymph node metastases, ruling out other concurrent systemic diseases (Figure 5). 

A follow-up CT scan five weeks after SBRT demonstrated further tumor regression (Figure 4d), with no recurrence of symptoms or acute treatment-related adverse events. Subsequently, the patient experienced low back pain following a fall at home. Although a follow-up CT scan revealed no acute traumatic injuries, such as fractures, she was admitted to our palliative care ward due to her living situation and to alleviate family anxiety. One week after admission, she was found dead during the night. Based on the clinical circumstances, her death was attributed to natural causes associated with advanced age. Her overall survival was 56 days from the initiation of SBRT for the lymph node metastasis (corresponding to 16 months after the initiation of CRT for the primary tumor).

## Discussion

In palliative care, pharmacotherapy alone for pain management cannot reduce tumor volume. As demonstrated in the present case, achieving tumor regression and alleviating pressure on surrounding tissues to preserve vital functions, such as swallowing and respiration, is crucial for maintaining the QOL, even in super-elderly patients. Radiotherapy is inherently less invasive than systemic chemotherapy or surgery. However, CRT poses challenges, including a high number of treatment fractions (which increases the burden of hospital visits) and adverse events in surrounding normal tissues. In particular, dermatitis, mucositis, dysphagia, and salivary gland dysfunction associated with irradiation of the head and neck region significantly compromise QOL. Desideri et al. reported that in PR for head and neck cancer, the most frequent Grade 3 acute toxicities were mucositis (7%) and dysphagia (15%), with late dysphagia also occurring in 7% of cases [10]. For patients with a limited life expectancy, the remaining lifespan may be insufficient to wait for either the onset of therapeutic efficacy or recovery from such severe acute adverse events. Therefore, PR for super-elderly patients requires a treatment strategy that delivers rapid therapeutic effects, minimizes adverse events, and shortens both the overall treatment duration and each fraction time.

SBRT is a highly effective palliative treatment modality owing to its precision targeting and superior dose distribution. Historically, stereotactic radiotherapy was predominantly utilized for cranial lesions-such as those managed with Gamma Knife-but recent technological advancements have expanded its application to extracranial sites, including the neck and trunk. In PR, regimens such as 8 Gy in a single fraction, 20 Gy in 5 fractions, and 30 Gy in 10 fractions are commonly employed; these fractionations are designed in accordance with the delivery principles of CRT [11]. CRT typically irradiates a relatively broad volume, including the normal tissues surrounding the lesion, using a lower dose per fraction. Although CRT simplifies treatment planning and delivery, the overall treatment period is prolonged due to the high number of fractions. Furthermore, the therapeutic effect is often gradual due to the relatively low intratumoral dose, and adverse events in adjacent normal tissues remain a major concern. Consequently, PR delivered via CRT typically requires several weeks to manifest clinical efficacy.

In contrast, depending on the treatment planning, SBRT can achieve rapid pain relief owing to its high intratumoral dose. Compared with CRT, SBRT can therefore provide more immediate symptom alleviation, a higher control rate, and a lower local recurrence rate [12]. Furthermore, because CRT involves large-field irradiation, the risk of adverse events increases significantly when the target volume overlaps with a previously irradiated field. Conversely, SBRT enables targeted delivery while shielding surrounding critical organs more effectively than CRT. In the present case, given that the patient had already undergone CRT for the primary tumor, SBRT was considered a highly appropriate treatment modality.

Systemic chemotherapy is occasionally considered even for super-elderly patients. However, several reports suggest that combining chemotherapy and radiotherapy in this population increases the risk of severe adverse events, and the indications must be carefully evaluated [13,14]. SBRT, being minimally invasive, requiring a short treatment duration, and offering the potential for rapid symptom relief, represents a promising option for palliative care in super-elderly patients. Nevertheless, radiotherapy remains a local therapy and does not inherently guarantee improved long-term survival or the prevention of distant metastases. Furthermore, SBRT typically requires a longer treatment planning and preparation period compared with CRT, which can occasionally delay the initiation of therapy. Although SBRT historically required longer single-fraction irradiation times than CRT, this limitation has been significantly mitigated by technological advancements such as FFF beams and VMAT [7].

In PR, treatment strategies must be tailored to individual patient profiles [4]. In the present case, to minimize the risk of adverse events-such as oral mucositis-and to maximize therapeutic efficacy, a heterogeneous dose distribution characterized by a high intratumoral dose and a steep dose fall-off at the target periphery was adopted [15]. Furthermore, to mitigate normal tissue toxicities, the treatment was delivered on alternate days [16]. Ultimately, an exceptionally early tumor reduction (biological response) and symptom alleviation were observed, exceeding our initial expectations. Consequently, we determined that the primary objectives of this palliative intervention had been fully achieved. To further shield the patient from potential adverse events, the scheduled third fraction was omitted. This type of situation-dependent ART represents a crucial strategy, particularly for super-elderly patients with reduced physiological reserve. Additionally, omitting the third fraction offered the secondary benefit of preserving normal tissue tolerance, thereby retaining the option for re-irradiation should local recurrence or new lesions emerge in the same region in the future.

Conversely, within the fields of home-based medical care and palliative care, the clinical utility of PR-particularly short-course, high-precision modalities such as SBRT-remains insufficiently recognized [17,18]. The present case was initiated via a direct request from a home-care physician with whom we had previously collaborated, representing a relatively uncommon referral pathway. Therefore, radiation oncologists must proactively disseminate clinical evidence and promote clinical awareness [19]. PR should not be perceived merely as a "last resort" after exhausting all other options; rather, it should be integrated as an appropriate component of palliative care from the early stages of diagnosis [20].

## Conclusions

Our case demonstrates the potential of advanced SBRT as an effective palliative care option in super-elderly patients. Because large-scale clinical trials in this specific population are challenging to conduct, a flexible, individualized assessment of treatment applicability remains essential. The observation period for this case was too short to adequately evaluate long-term treatment efficacy or late toxicity. Further accumulation of clinical experience with PR for super-elderly patients is warranted. Concurrently, radiation oncologists must widely disseminate the clinical utility and benefits of PR to the broader medical community.
